# Perceptions and reasons of E-cigarette use among medical students: an internet-based survey

**DOI:** 10.1186/s42506-020-00051-0

**Published:** 2020-08-17

**Authors:** Abdulmohsen A. Alzalabani, Sheref M. Eltaher

**Affiliations:** 1 Family and Community Medicine Department, Faculty of Medicine, Taibah University, Madinah, Kingdom of Saudi Arabia; 2 Public Health and Community Medicine Department, Faculty of Medicine, Benha University, Benha, Egypt

**Keywords:** E-cigarette, Reasons, Perception, Taibah University, KSA

## Abstract

**Background:**

Nowadays, E-cigarette use shows a significant increase among adults and youth in many countries, although there is still a public health debate about its relative effects compared to the traditional cigarettes.

**Objectives:**

To assess the prevalence, perceptions, possible reasons of use, and factors associated with E-cigarettes use among medical students at Taibah University, Kingdom of Saudi Arabia.

**Methods:**

An internet-based online survey study was conducted between September and November 2019 which contains socio-demographic data, data related to smoking and E-cigarette use, and data related to reasons of E-cigarette use.

**Results:**

A total of 527 out of 720 students completed an online questionnaire, 15.9% of the surveyed students used E-cigarettes, older age, male, higher college class, those who have ≥ 1 close friend who smokes, family history of smoking, and housemates who smoked E-cigarettes show a significantly higher percentage of E-cigarettes use. The first reason for E-cigarettes use namely that, E-cigarette helps to reduce tobacco consumption with no intention to quit smoking was the highest (89.2%), followed by E-cigarette being less toxic than tobacco (88.4%), and finally E-cigarette helps to avoid having to go outside to smoke (62.05%). Logistic regression analysis showed that sex, more than one close friend who smokes, family history of smoking, and housemates who smoked E-cigarettes were significant factors associate with its use.

**Conclusion:**

E-cigarette use appears to be relatively common than conventional cigarette among the participants. Our study participants perceived that it can help people quit, less harmful, and less addictive. The most common reason for its use among the study participants was that E-cigarette helps to reduce tobacco consumption in users with no intention to quit smoking.

## Introduction

Electronic nicotine delivery systems (ENDS) (E-cigarettes, vapes) are battery-provided devices in which nicotine is delivered to users in the form of vapor [1]. E-cigarettes were introduced in the markets since mid-to-late 2000s. Nowadays, there is an increase in the use of E-cigarettes by youth and adults [2].

The use of E-cigarettes and its public health effect are still debatable, as it is believed by some people that it can reduce tobacco cigarettes harm in people who cannot quit smoking, while others think it is the gateway for cigarette smoking [3]. Laboratory testing for some E-cigarette cartridges found that it may contain some toxic and little amount of carcinogenic components [4].

E-cigarette may be used because smokers/people either want to quit or want to continue smoking in public places. E-cigarettes are considered an alternative for ordinary cigarettes which are thought to be more harmful and more expensive [5].

It has been noticed that the use of E-cigarette has remarkably increased in the last few years specially in young people [6]. There is remarkable variation in prevalence of E-cigarette among youth from country to another. E-cigarette ever use was highest in Poland (62.1%) and lowest in Italy (5.9%), while in non-smoking youth, the prevalence ranged from 14% in New Zealand to 4.2% in the USA. The highest prevalence among tobacco smokers was in Canada (71.9%) while the lowest was in Italy (29.9%) [7]. Centers for Disease Control and Prevention (CDC) reported the first case of E-cigarette, or vaping, product use-associated lung injury (EVALI) [8, 9].

According to the WHO, long-term health effects of ENDS are still unknown; although the aerosol that they inhale contains toxic substances that may increase the risk of cancer or cardiovascular or pulmonary disease [10]. However, the National Academies of Sciences, Engineering, and Medicine stated that E-cigarette aerosol contains fewer numbers and lower levels of toxicants than smoke from combustible tobacco cigarettes [11].

Our objective is to assess the prevalence, perceptions, possible reasons of use and factors associated with use of E-cigarettes among medical students at Taibah University, Kingdom of Saudi Arabia.

## Methods

### Study design

An internet-based online survey study was conducted between September and November 2019 in College of Medicine, Taibah University, Kingdom of Saudi Arabia (KSA).

### Study participants

All the undergraduate students in the College of Medicine, Taibah University, KSA, whose total number was 720 students were targeted.

### Recruitment of the study participants

The participants were invited to participate in the survey through email containing a link to the online survey and also through the male and female leaders of each study year. The online questionnaire was done through google e-forms, and every student was allowed to give only one response. Out of 720 students, 590 answered the questionnaire with a response rate of 82% and 63 students were excluded from the study due to incomplete responses; thus, the total number after their exclusion was 527 students, and there was no statistical difference between those who completed and those who did not complete the questionnaire regarding the collected data.

### Data collection tool

An online questionnaire was composed of:

i) Socio-demographic data (age, sex and college class which is either first two years (pre-clinical) or last 3 years (clinical)),
ii) Data related to smoking and E-cigarette use (type of smoking, family history of smoking, ≥ 1 close friend who smokes, housemates who smoked E-cigarettes, and perception were operationally defined as students’ responses to the following three statements: whether E-cigarettes can help people quit, E-cigarettes are less harmful than tobacco cigarettes and E-cigarettes are less addictive than cigarettes.
iii) Data related to reasons of E-cigarette use (E-cigarette is less toxic than tobacco, E-cigarette helps to deal with craving for tobacco, E-cigarette helps to quit smoking or avoid relapsing, E-cigarette helps to deal with withdrawal symptoms, E-cigarette is cheaper than smoking, E-cigarette helps to avoid nuisance of others with tobacco smoke, E-cigarette helps to deal with situations where one cannot smoke, E-cigarette helps to avoid having to go outside to smoke, E-cigarette helps to reduce tobacco consumption in preparation of a quit attempt, and E-cigarette helps to reduce tobacco consumption with no intention to quit smoking).

The online survey composed of 2 sections: the first one contained 11 question and the second one contained 10 questions; all questions were multiple choice questions, and it takes about 10––15 min to be completed.

The questionnaire was designed by the researchers and was adapted from previous relevant literature. The questionnaire was in English. Its contents were validated by a consultant pulmonologist and pre-tested on 10 students as a pilot study which was not included in the results. Relevant modifications were instituted prior to commencement of actual data collection. Cronbach’s alpha for the questionnaire was at the level of 0.71.

### Statistical analysis

Data were tabulated, coded, and analyzed using the Statistical Package for the Social Sciences (SPSS) software version 20.0 for Windows. Quantitative data as age was summarized with mean and standard deviation. Qualitative data was expressed in frequencies and percentage. Chi-square “χ2” test was used to compare categorical data. Odds Ratio and 95% confidence interval was calculated. Logistic regression analysis was done to detect factors associated with E-cigarette use, and only factors which were significant in the univariate analysis were entered. A *p* value of < 0.05 was considered significant.

## Results

Table 1 shows the characteristics of 527 students who completed the questionnaire: their mean age was 22.2 ± 2.3 years old, 59.8% of them were males, 51.6% were in the clinical years (year 3, 4, and 5), 15.9%, 15.7% and 5.7% of the surveyed students used E-cigarettes, conventional smoking and mixed type respectively (not shown in Table 1), 44.2% of them had ≥ 1 close friend who were smokers, 74.4% had positive family history of smoking, and 70.6% have housemates who smoked E-cigarettes. Regarding perception of E-cigarette, 40% of the surveyed students agreed that E-cigarettes can help people quit, while 30.6% agreed that E-cigarettes are less harmful than cigarettes and 45.9% agreed that E-cigarettes are less addictive than cigarettes.

**Table 1:** Comparison between E-cigarette users and non-users regarding demographic characteristics and perception of E-cigarette, Medical College, Taibah University, KSA (*n* = 527)

		E-cigarette users (*n* = 114)		E-cigarette non-users (*n* = 413)		Total		*χ2*	OR (95% CI)	*p* value
		No.	%	No.	%	No.	%			
Age (years)	18	37	32.46	186	45.04	223	42.3	5.8	1.7 (1.1–2.6)	0.02*
	21–25	77	67.54	227	54.96	304	57.7			
Sex	Female	11	9.7	201	48.7	212	40.2	56.7	8.9 (4.6–17.02)	< 0.001*
	Male	103	90.3	212	51.3	315	59.8			
College class	Pre-clinical years	42	36.8	213	51.6	255	48.4	7.8	1.9 (1.2–2.8)	0.005
	Clinical years	72	63.2	200	48.4	272	51.6			
≥ 1 close friend smokes	Yes	93	81.6	140	33.9	233	44.2	82.3	8.6 (5.2–14.5)	< 0.001*
	No	21	18.4	273	66.1	294	55.8			
Family history of smoking	Yes	98	85.9	294	71.2	392	74.4	10.2	2.5 (1.4–4.4)	0.001
	No	16	14.1	119	28.8	135	25.6			
Housemates who smoked E-cigarettes	Yes	89	78.1	283	68.5	372	70.6	3.9	1.6 (1.002–2.67)	0.047
	No	25	21.9	130	31.5	155	29.4			
Perception about E-cigarette										
E-cigarettes can help people quit	Strongly agree or agree	50	43.9	161	38.9	211	40	2.01	0.7 (0.4–1.1)	0.2
	Undecided	37	32.5	83	20.1	120	22.8			
								13.04	0.4 (0.2–0.6)	< 0.001*
	Disagree or strongly disagree	27	23.6	169	41.1	196	37.2			
E-cigarettes are less harmful than cigarettes	Strongly agree or agree	65	57	96	23.2	161	30.6	12.9	2.4 (1.4–3.9)	< 0.001*
	Undecided	36	31.6	128	30.9	164	31.1			
								18.8	0.2 (0.1–0.5)	< 0.001*
	Disagree or strongly disagree	13	11.4	189	45.9	202	38.3			
E-cigarettes are less addictive than cigarettes	Strongly agree or agree	60	52.6	182	44.1	242	45.9	0.06	1.06 (0.6–1.7)	0.8
	Undecided	32	28.1	103	24.9	135	25.6			
								3.8	0.6 (0.3–1.01)	0.052
	Disagree or strongly disagree	22	19.3	128	31	150	28.5			

* significant at *p* <0.05

** significant at *p* < 0.001

There was a statistically significant difference between E-cigarette users and non-users regarding age (*p* = 0.02), sex (*p* < 0.001), college class (*p* = 0.001), ≥ 1 close friend smokes (*p* < 0.001), family history of smoking (*p* = 0.001), and housemates who smoked E-cigarettes (*p* = 0.047).

The group aged 21–25 years old are 1.7 times more likely to use E-cigarette than younger group, males are 8.9 times than females, clinical years’ students are 1.9 times than pre-clinical years, those who had ≥ 1 close friend who smokes are 8.6 times than who had not, students with positive family history of smoking are 2.5 times more than who had negative family history, and students who had housemates who smoked E-cigarettes are 1.6 times more than those who had not.

On comparison of perception of E-cigarette, more E-cigarette users agreed that E-cigarettes can help people quit compared with non-users (43.9% versus 38.9%). A significantly higher proportion of users of E-cigarettes agreed that E-cigarettes are less harmful than cigarettes compared with the non-users (57% versus 23.2%, *p* < 0.01). Likewise, users of E-cigarettes were more likely to perceive the E-cigarettes as being less addictive than non-users (52.6% versus 44.1%). However, this difference was not statistically significant.

The reasons of using E-cigarette among the surveyed students are presented in Table 2. The most frequently reported reason was that E-cigarette helps to reduce tobacco consumption with no intention to quit smoking (89.2%). This was followed by E-cigarette being less toxic than tobacco (88.4%) while the last reason was that E-cigarette helps to avoid having to go outside to smoke (62.05%).

**Table 2:** Reasons for using E-cigarette among surveyed students, Taibah University, KSA (*n* = 527)

Reasons	E-cigarette users (*n* = 114)		E-cigarette non-users (*n* = 413)		Total		*χ2*	*p* value
	No.	%	No.	%	No.	%		
1. E-cigarette is less toxic than tobacco	93	81.6	373	90.3	466	88.4	6.7	0.009*
2. E-cigarette helps to deal with craving for tobacco	97	85.1	329	79.7	426	80.8	1.7	0.2
3. E-cigarette helps to quit smoking or avoid relapsing	73	60.1	297	71.9	370	70.2	2.7	0.1
4. E-cigarette helps to deal with withdrawal symptoms	87	76.3	301	72.9	388	73.6	0.5	0.5
5. E-cigarette cheaper than smoking	22	19.3	311	75.3	333	63.2	120.5	< 0.001**
6. E-cigarette helps to avoid bothering others with tobacco smoke	101	88.6	343	83.1	444	84.3	2.1	0.2
7. E-cigarette helps to deal with situations where one cannot smoke (at work, etc.)	57	50	291	70.5	348	66.03	16.7	< 0.001**
8. E-cigarette helps to avoid having to go outside to smoke	49	42.9	278	67.3	327	62.05	22.5	< 0.001**
9. E-cigarette helps to reduce tobacco consumption in preparation of a quit attempt	87	76.3	378	91.5	465	88.2	19.9	< 0.001**
10. E-cigarette helps to reduce tobacco consumption with no intention to quit smoking	79	69.3	391	94.7	470	89.2	59.6	< 0.001**

* significant at *p* < 0.05

** significant at *p* < 0.001

Comparing the other perceived merits, there was statistically significant differences between E-cigarette users and non-users regarding the following, E-cigarette being less toxic than tobacco, E-cigarette being cheaper than smoking, E-cigarette helping to deal with situations where one cannot smoke (at work, etc.), E-cigarette helping to avoid having to go outside to smoke, E-cigarette helping to reduce tobacco consumption in preparation of a quit attempt, and E-cigarette helping to reduce tobacco consumption with no intention to quit smoking.

Regression analysis on factors associated with E-cigarettes use revealed that sex (*p* < 0.001), ≥ 1 close friend smokes (*p* < 0.001), family history of smoking (*p* = 0.001) and housemates who smoked E-cigarettes (*p* = 0.031) were significant factors associated with use while age and college class were non-significant factors (Table 3).

**Table 3:** Factors associated with using E-cigarettes among surveyed students, Taibah University, KSA (*n* = 527)

	AOR^#^ of E-cigarette use (95% CI)	*p* value
Age	1.3 (0.6–1.9)	0.07*
Sex	6.9 (3.9–14.7)	< 0.001**
College class	0.43 (0.23–1.09)	0.06*
≥ 1 close friend smokes	9.2 (4.3–15.8)	< 0.001**
Family history of smoking	2.9 (1.7–5.1)	0.001**
Housemates who smoked E-cigarettes	2.2 (1.3–3.7)	0.031*

^#^*AOR* adjusted odds ratio

* significant at *p* < 0.05

** significant at *p* equal to or < 0.001

## Discussion

The present study showed that the prevalence of cigarette smoking among the studied cohort was 15.7%, that of E-cigarette was 15.9%, and mixed smoking was 5.7%. The prevalence of cigarette smoking is comparable to the previous study done in Jeddah among health science students (14.1%) [12] and also that reported by WHO 2019 (12.2%) [13]. The prevalence of E-cigarette was comparable to other studies in Saudi Arabia as it was slightly lower than a study in Jeddah [12] but slightly higher than a study in Al-Qassim [14]. The prevalence of E-cigarettes was 25% in England and that among USA young adults was 20.8% [15]. However, a study in the USA that an estimated 27.5% of high school students reported E-cigarette use [16]. It was higher than that of cigarette smoking in our participants. This may be due to increase in availability of E-cigarette nowadays and the belief of being allegedly less harmful. Lower prevalence in the present survey compared to western countries may be attributed to the effect of tradition in our communities.

E-cigarette use among participants in our study was more prevalent in older age group, male students, higher college class, students who have ≥ 1 close friend who smokes, family history of smoking, and housemates who smoked E-cigarettes. This is partially in accordance with Canzan et al. who stated that E-cigarette use was associated with housemates*’* smoking habits, male sex but not with a family history of smoking and university class [17]; but it is not in agreement with Jiang et al. who found that there was non-significant difference between males and females regarding E-cigarette use [18]. It is in agreement with Almutham et al. who stated that E-cigarette smoking had a significant correlation with having a family member or a close friend who smokes E-cigarettes [19]. This may be explained by that the pro E-cigarette social environment represented a very important incentive to start using E-cigarettes.

Regarding perception about E-cigarettes, participants of the present study mostly agreed that E-cigarettes can help people quit, it is less harmful and less addictive than cigarettes, and these thoughts are more among E-cigarette users. This is partially agreeing with Almutham et al. who found that medical students believe that E-cigarette are less harmful and less addictive than traditional cigarette smoking; however, most of medical students in that study did not agree that E-cigarettes could help patients quit smoking [19]. Moreover, this is in agreement with a study done in Hong Kong about perceptions and use of E-cigarettes among young adults in which participants stated that E-cigarettes were perceived as less harmful and less addictive [18]. This misconception may be due to the fact that students in our study did not receive any form of education about E-cigarettes and also had less confidence to discuss the matter of E-cigarettes with their parents and until now there is a lot of misconception about its health risk among some physicians.

The most frequent reasons for using E-cigarette among our surveyed students were E-cigarette helps to reduce tobacco consumption with no intention to quit smoking and E-cigarette is less toxic than tobacco. Etter and Bullen noticed in their study that the reasons for using E-cigarette were the perception that it was less toxic than tobacco, to deal with craving for tobacco and withdrawal symptoms, to quit smoking or avoid relapsing, being cheaper than smoking, and to deal with situations where smoking was prohibited [20]. Another study done by Rhoades et al. showed that the most common reasons for E-cigarette vaping among adults and adolescents were to decrease smoking, and the ability to use it in non-smoking places, while posing less harm to other people or to oneself in comparison to conventional smoking were the next most frequent responses. Less than one-half used E-cigarette to cut down on stress, lessen cost, or because of using E-cigarette by important persons to them [21]. It was noticed that the common shared causes of E-cigarette use mentioned in different studies was its being less harmful and toxic, it helps in quitting smoking and the practicality of its use in the areas where smoking is prohibited.

On determining factors associated with E-cigarettes use, this study revealed that sex, ≥ 1 close friend smokes, family history of smoking, and housemates who smoked E-cigarettes were significant factors associated with its use. This is in accordance with a study done by Romijnders et al. in which social environment, for example a friend, a father, or a mother who uses E-cigarette, was an initiator for E-cigarette use [21]. Another study stated that E-cigarette use is affected by sex but not affected by age [17]. This supports the concept that any family member or friend (social environment) who uses E-cigarette is an initiative for starting its use.

### Study limitations

This study is an online survey targeting specific group of population and confined to one geographical area which affects generalization of results. Moreover, the study questionnaire did not include certain items like traditional cigarette smokers and other factors like social class and educational level of parents. So, further large follow-up studies should be done to explore the prevalence, health effects, and factors affecting using E-cigarette especially at community level.

## Conclusion

E-cigarette use appears to be relatively common than conventional cigarette among the participants. Our study participants perceived that it can help people quit, less harmful, and less addictive. The most common reason for its use among the study participants was that E-cigarette helps to reduce tobacco consumption with no intention to quit smoking

## Data Availability

Data can be made available from the corresponding author upon request and with the permission of authorities. All authors had full access to all data in the study and take responsibility for the integrity of the data and the accuracy of the data analysis.

## Abbreviations

AOR: Adjusted odds ratio
CDC: Centers for Disease Control and Prevention
E-cigarettes: Electronic -cigarettes
ENDS: Electronic nicotine delivery systems
EVALI: E-Cigarette, or vaping, product use-associated lung injury
KSA: Kingdom of Saudi Arabia
WHO: World Health Organization
